# A novel m7G methylation–related signature associated with chromosome homeostasis in patients with lung adenocarcinoma

**DOI:** 10.3389/fgene.2022.998258

**Published:** 2022-10-24

**Authors:** Xiaoying Tao, Run Huang, Rujun Xu, Shuang Zheng, Juanqing Yue

**Affiliations:** ^1^ Department of Pathology, Affiliated Hangzhou First People’s Hospital, Zhejiang University School of Medicine, Hangzhou, China; ^2^ Department of Infectious Diseases, Shanghai Jiao Tong University Affiliated Sixth People’s Hospital, Shanghai, China

**Keywords:** lung adenocarcinoma, m7G methylation, gene signature, chromosome homeostasis, immune station

## Abstract

Lung adenocarcinoma (LUAD) is a malignant tumor of the respiratory system with poor prognosis. Recent studies have revealed that N7-methylguanosine (m7G) methylation is a widespread modification occurring in RNA. But the expression of m7G methylation–related genes in LUAD and their correlations with prognosis are still unclear. In this study, we found 12 m7G methylation–related regulators with differential expression between LUAD and normal lung tissues. According to differentially expressed genes (DEGs), all LUAD cases were separated into two subtypes. The prognostic value of each m7G methylation–related gene for survival was evaluated to construct a multigene signature using The Cancer Genome Atlas (TCGA) cohort. Finally, an m7G methylation–related prognostic signature based on three genes was built to classify LUAD patients into two risk groups. Patients in the high-risk group showed significantly reduced overall survival (OS) when compared with patients in the low-risk group (*p* < 0.05). The receiver operating characteristic (ROC) curve analysis confirmed the predictive capacity of the signature. The Gene Ontology (GO) functional annotation analysis disclosed that chromosome homeostasis plays an important role in this process. The gene set enrichment analysis (ssGSEA) implied that the immune status was decreased in the high-risk group. To sum up, m7G methylation–related genes play a vital role in tumor immunity and the related signature is a reliable predictor for LUAD prognosis.

## Introduction

Lung adenocarcinoma (LUAD) is the most common histological type of lung cancer. Despite advances in its pathogenesis and therapeutic approaches, it unfortunately remains one of the most aggressive and fatal tumors with an overall survival (OS) of less than 5 years (Denisenko et al., 2018). Therefore, it is important to understand the underlying molecular mechanisms and develop an accurate prognostic tool to improve the progression of LUAD.

To date, with a large number of RNA modifications, N7-methylguanosine (m7G) has been found to be the most common modification at the 5′ cap of mRNA and exert an important part in the multicellular processes and regulation of mRNA output, translation, transcriptional elongation, and splicing. METTL1/WDR4 has been identified as an m7G writer of mRNA. METTL1 acts as an m7G methyltransferase to install m7G modifications in target mRNAs, while WDR4 facilitates the binding of heterodimeric complexes to target mRNAs (Zhang et al., 2019a). Accumulating evidence point to the critical role of m7G in human disease development, especially cancer, and aberrant m7G levels are closely related to tumorigenesis and progression by regulating the expression of multiple oncogenes and tumor suppressor genes (Luo et al., 2022). Interestingly, various studies to date have shown that m7G modulators play different roles in different types of tumors. METTL1/WDR4 is the core regulator of m7G modification and exerts a powerful oncogenic role in acute myeloid leukemia (AML) (Orellana et al., 2021), bladder cancer (BC) (Ying et al., 2021), esophageal squamous cell carcinoma (ESCC) (Han et al., 2022), glioma (Li et al., 2021a), hepatocellular carcinoma (HCC) (Tian et al., 2019; Chen et al., 2021; Xia et al., 2021), head and neck squamous cell carcinoma (HNSCC) (Chen et al., 2022), etc., promoting the malignant phenotype and progression of tumors. However, a recent study investigated the functions of m7G regulators WBSCR22 and TRMT112 in pancreatic cancer (PC) and found that WBSCR22 was downregulated in PC samples when compared with adjacent normal pancreatic tissue and WBSCR22 cooperates with TRMT112 to exert a tumor suppressor effect in PC, associated with longer survival of patients (Khan et al., 2022).

Although evidence shows RNA modifications are critical to the development and progression of LUAD, few researchers have systematically explored the relationship between m7G methylation and LUAD. In this study, we systematically assessed the prognostic value of m7G regulatory genes for LUAD patients. The expression profiles and clinical information were obtained from the TCGA database. Additionally, online databases such as the R software package were used for bioinformatics analysis to study the characteristics of m7G regulatory factors. Besides, we assessed the association of the prognostic model based on m7G regulatory genes with survival outcomes, immune-related pathways, and immune cell infiltration. We found that m7G was involved in several aspects of LUAD, such as the formation of tumor microenvironment (TME). A better understanding of m7G modifications makes it possible to develop more effective personalized treatment strategies for LUAD.

## Materials and methods

### Data sets

In this study, we downloaded the RNA sequencing information and the corresponding clinical characteristics of LUAD patients from the TCGA database (https://portal.gdc.cancer.gov/), which included 535 tumors and 59 normal tissues (Cancer Genome Atlas Research Network and others, 2014; Campbell et al., 2016). The expression data sets could be normalized by fragment per kilobase million (FPKM) scores.

### Identifying differentially expressed m7G methylation–related genes

We curated a total of 29 m7G methylation–related genes (Supplementary Table S1) by reviewing previous literature (Tomikawa, 2018; Rong et al., 2021; Wiener and Schwartz, 2021). These m7G methylation–related genes were studied using the Search Tool for the Retrieval of Interacting Genes (STRING). Using the “limma” R package, we identified differentially expressed m7G methylation–related genes among tumor and normal tissues with a false discovery rate (FDR) within 0.05 and |logFC| > 0.5.

### Development and validation of m7G methylation–related gene signature

The consensus cluster method was adopted to construct the consensus matrix according to the expression levels of the differentially expressed genes (DEGs). We evaluated clustering results with K values ranging from 2 to 9 in order to determine the optimal clustering results. A univariate Cox study of the OS was used to screen DEGs using prognostic values [adjusted *p*-value <0.05; *p*-values were adjusted using Benjamini and Hochberg (BH) correction]. Then, the DEGs with prognostic values were applied to construct a signature in prognostic prediction. By combining standard expressions of every gene and its relevant regression coefficients, the risk scores were calculated as follows:

$$\text{Risk}\;\text{Score}=\sum_{i=1}^{n}Coefi\times xi.$$

LUAD in TCGA could be regulated as two risk groups (high/low) based on the median value of the risk score. According to the gene expression in this signature, the principal component analysis (PCA) was conducted by means of the “prcomp” function of the R statistical package. The prognostic model of genes was assessed by the time-dependent receiver operating characteristic (ROC) profile analyses *via* the “survivalROC” R package. The nomogram was built by combining age, gender, risk scores, and pathology stages to forecast the living possibility of LUAD patients after 3 and 5 years. The Decision Curve Analysis (DCA) was carried out to evaluate the clinical implications of the prediction model.

### Gene Ontology functional and immune analysis of differentially expressed genes among low- and high-risk groups

As aforementioned, LUAD patients were regulated as two risk groups based on the median value. The DEGs among the high-risk and low-risk groups could be determined with |log2FC| ≥ 1 and FDR <0.05. The GO annotation analysis of DEGs was achieved with an adjusted *p*-value < 0.05. Accumulating evidence have confirmed that tumor infiltrating immune cells were involved in cancer progression and correlated with the outcomes. Therefore, ssGSEA was used to estimate the permeation values for immunization-related cells and analyze the activity of immunization-related pathways. Supplementary Table S2 provides these annotated gene set files. Meanwhile, we conducted correlation analysis between immune cells and immune functions and between genes for signature construction and immune cells and immune function. Furthermore, we evaluated the infiltration level of immune cells between the high-risk and low-risk groups by using CIBERSORT, CIBERSORT-ABS, quanTIseq, MCP-counter, xCell, TIMER, and EPIC algorithms.

### Statistical analysis

**FIGURE 1:**
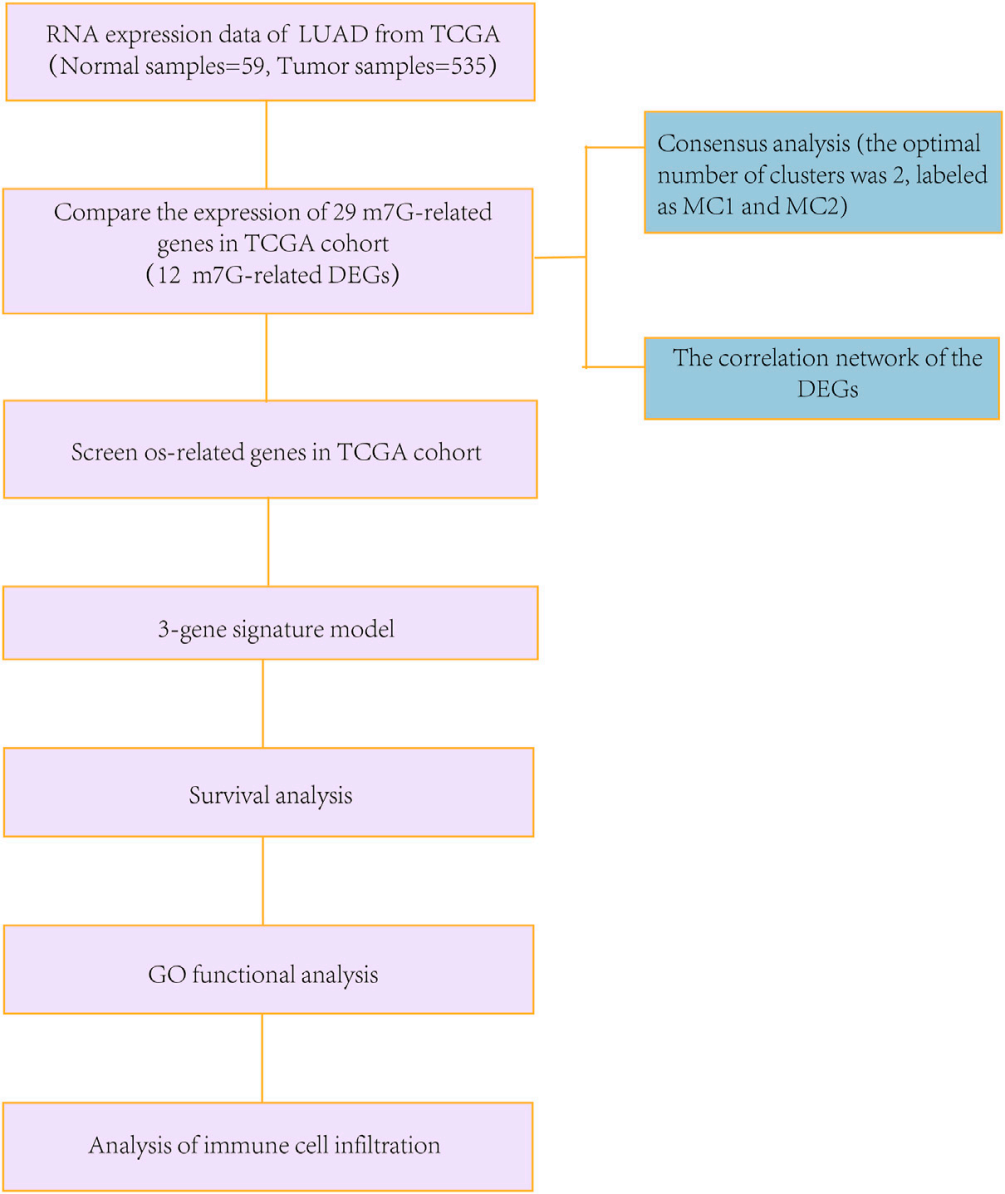
Detailed workflow of the flowchart for data analysis.

To compare the standards of gene expression among normal and LUAD tissues, a single-factor analysis of variance was used, and the Pearson’s Chi-square test was used to contrast the classified variances. Nomogram was constructed with the “RMS” R package. BH-adjusted Mann–Whitney tests were conducted to compare the ssGSEA scores between the high- and low-risk subgroups of immune cells or pathways. Kaplan–Meier analysis using log-rank tests were used to contrast the OS among different groups. Data analyses were carried out using the R software (v. 4.1.2). The flowchart is shown in Figure 1.

## Results

### m7G methylation–related differentially expressed genes in The Cancer Genome Atlas lung adenocarcinoma queue

**FIGURE 2:**
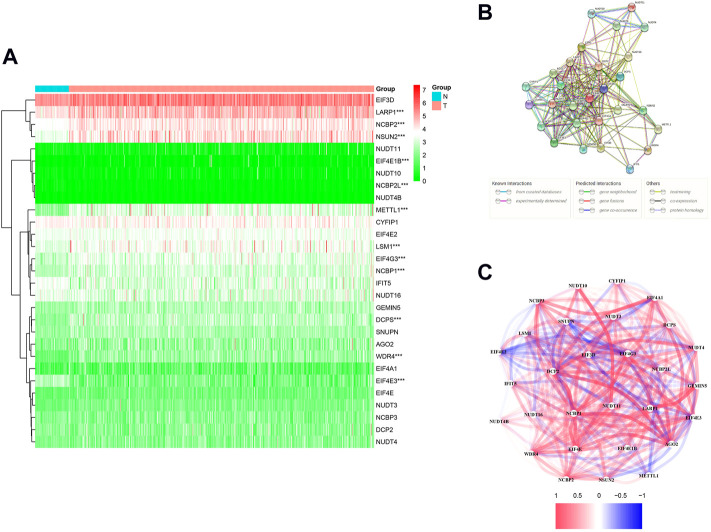
Expression and interaction of 29 m7G methylation–related genes. **(A)** Heatmap (green: low expression; red: high expression) of m7G methylation–related genes between normal (N, bright blue) and tumor (T, pink) tissue in the TCGA LUAD queue. **(B)** PPI network shows interactions of all m7G methylation–related genes. **(C)** Correlation network of all m7G methylation–related genes (red: positive relevance; blue: negative relevance. The shade of color reflects the strength of the correlation). *p < 0.05, **p < 0.01; * * *p < 0.001.

We identified 12 m7G-related DEGs (FDR < 0.05) by comparing the expression levels of all m7G methylation–related genes in LUAD tissues (*n* = 535) with normal tissues (*n* = 59) in the TCGA database. The mRNA levels of all m7G methylation–related genes are presented as heatmaps (Figure 2A). The protein–protein interaction (PPI) network and gene correlation network showed the interaction relationships of all m7G methylation–related genes (Figures 2B,C).

### Construction of three m7G methylation–related prognostic model in The Cancer Genome Atlas queue

**FIGURE 3:**
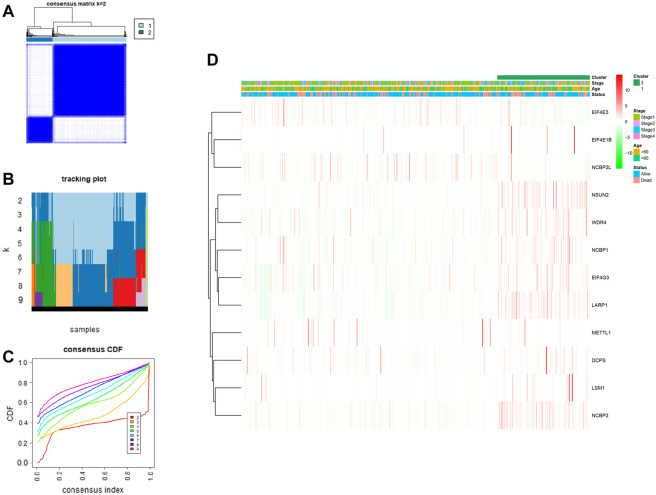
LUAD classification according to m7G methylated–associated DEGs. **(A–C)** Consensus clustering algorithm applied to cluster LUAD samples in the TCGA database. The best number of clusters was two, which are defined as MC1 and MC2, respectively. **(D)** Heatmap and clinicopathological characteristics of two clusters.

**FIGURE 4:**
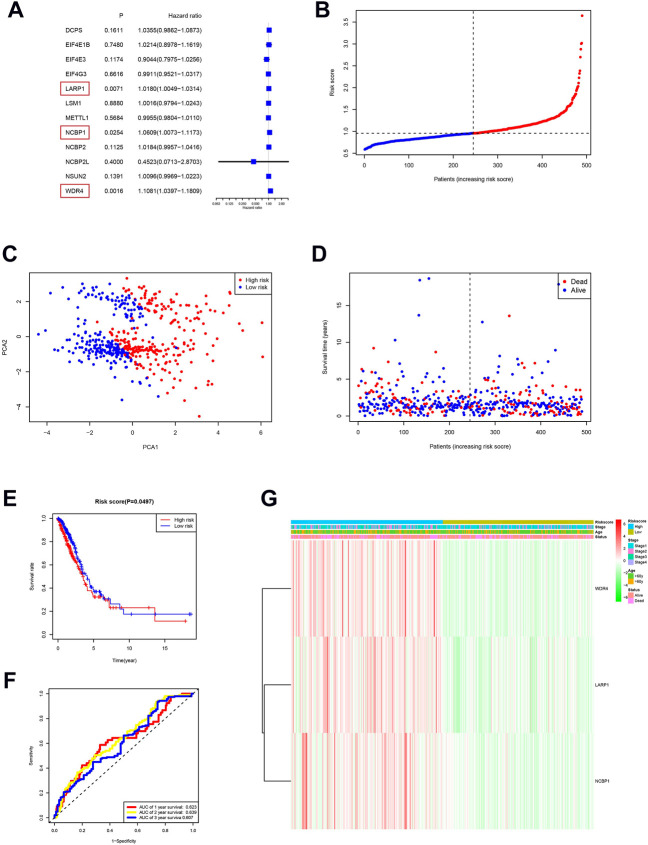
Risk signature establishment of the TCGA queue. **(A)** Single-variable Cox regression study of 12 m7G methylation–related DEGs and three genes with *p* < 0.05. **(B)** Patient distribution on the basis of risk score. **(C)** PCA diagram of LUADs on the basis of risk score. **(D)** Living situation of every patient (low-risk population: left dotted line; high-risk groups: right dotted line). **(E)** Kaplan–Meier survival analysis for patients with high- and low-risk groups. **(F)** ROC profile showing the forecasting power of risk scores. **(G)** The relationship among clinicopathological characteristics and risk groups is visualized with heatmaps (green: low expression; red: high expression).

**FIGURE 5:**
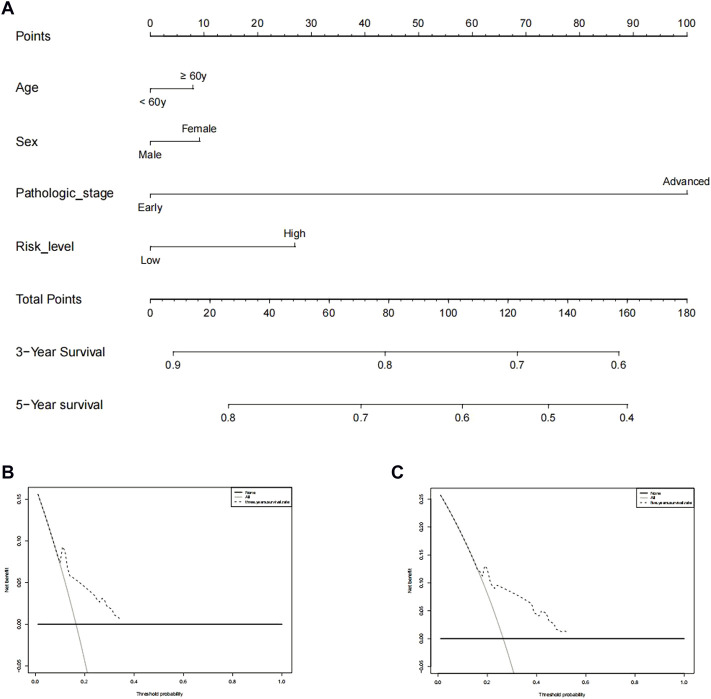
Prediction of 3- and 5-year survival probabilities for nomogram LUAD patients. **(A)** An upward line is drawn from these values to a “score” line based on age (≥60 or <60 years), sex, pathological stage (early stages include stages 1 and 2, advanced stages include stages 3 and 4), and risk levels. Points are calculated by drawing an upward vertical line on the “points” column. Adding these “points” gives the “total point” and a vertical line is drawn down from the “total point” line to count the 3-year and 5-year living probabilities. **(B,C)** Decision curve analysis of multi-Cox models for 3 and 5 years, respectively.

We performed a consensus clustering analysis of all 535 LUAD from the TCGA cohort to investigate the relationship among expressions of 12 m7G methylation–related DEGs and the subtypes of LUAD. Adding the clustering variable (k) from 2 to 9, when *k* = 2, showed the highest intragroup relevance and the lowest intergroup relevance, hinting that the optimal number of clusters was two, defined as MC1 and MC2 (Figures 3A–C). The profile of the DEG expressions and clinical features such as age (≤60 or >60 years), the degree of cancer stage (stages 1–4), and living status (alive or dead) are shown in the heatmap (Figure 3D). The univariate Cox regression analysis identified three DEGs that were correlated with the OS, namely, LARP1, NCBP1, and WDR4 (Figure 4A). Therefore, these three DEGs were used to construct the risk model. Further multivariate Cox proportional hazards analysis suggested that all the three DEGs were related to increasing risk with HRs >1 (Supplementary Table S3). The formula used for risk value calculation is risk value = (0.0113 × LARP1 exp.) + (0.0255 × NCBP1 exp.) + (0.0817 × WDR4 exp.). The LUAD patients in the TCGA were separated into the high-risk group (*n* = 262) or low-risk group (*n* = 248) with a middle score (Figure 4B). The PCA showed that the different risk groups could be well divided into two clusters (Figure 4C), and patients in the high-risk group survived less and lived shorter than did those in the low-risk group. Consistently, the Kaplan–Meier profile indicated that the OS time difference between the low- and high-risk groups was statistically significant, and the high-risk group had worse OS than their low-risk counterparts (Figures 4D,E). Time-dependent ROC analysis was performed, and the area under the ROC curve (AUC) was 0.623, 0.639, and 0.607 for a 1-, 2-, and 3-year survival, respectively (Figure 4F). The clinicopathologic features, along with the associated risk groups, were mapped using a heatmap (Figure 4G). The nomogram was constructed for forecasting the 3- and 5-year living probability of LUAD patients (Figure 5A). The DCA for the 3- and 5-year prediction of m7G-related gene signature nomogram is illustrated in Figures 5B,C.

### Functional analysis according to risk model

**FIGURE 6:**
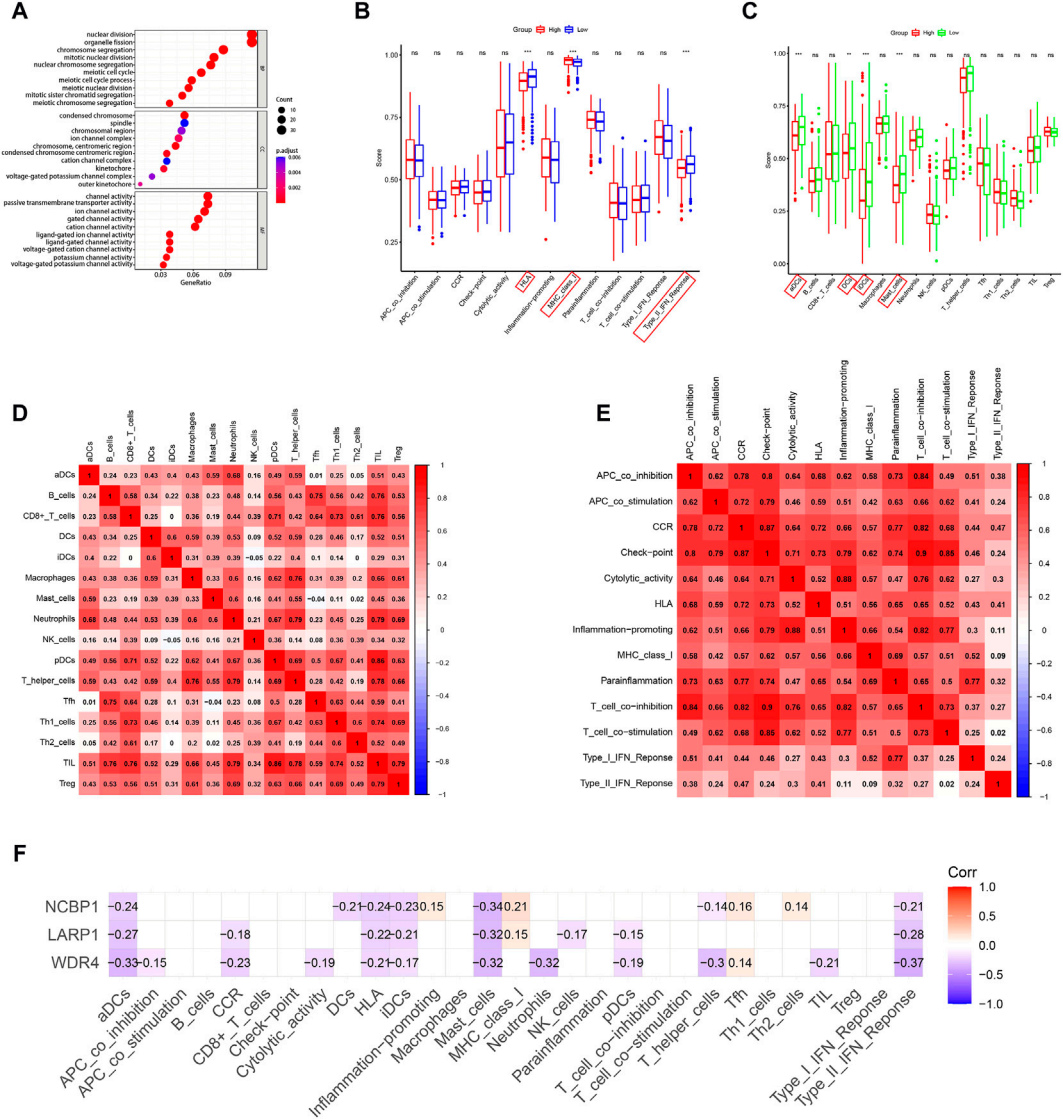
GO and immune analyses according to DEGs among two risk groups in the TCGA queue. **(A)** Bubble graph for GO functional annotation analysis (the smaller bubble means fewer genes are enriched, and the increasing depth of red means the differences are more obvious). **(B)** By contrast, enrichment fractions of 16 kinds of immune cells in the low- (bule box) and high-risk (red box) groups. **(C)** By contrast, enrichment values of 13 immune-related pathways among low-risk (green box) and high-risk (red box) groups. Correlations between **(D)** immune cells and **(E)** immune functions in LUAD patients. **(F)** Correlation analysis of *WDR4*, *LARP1*, and *NCBP1* with immune cells and immune function. *p < 0.05; **p < 0.01; ***p < 0.001.

**FIGURE 7:**
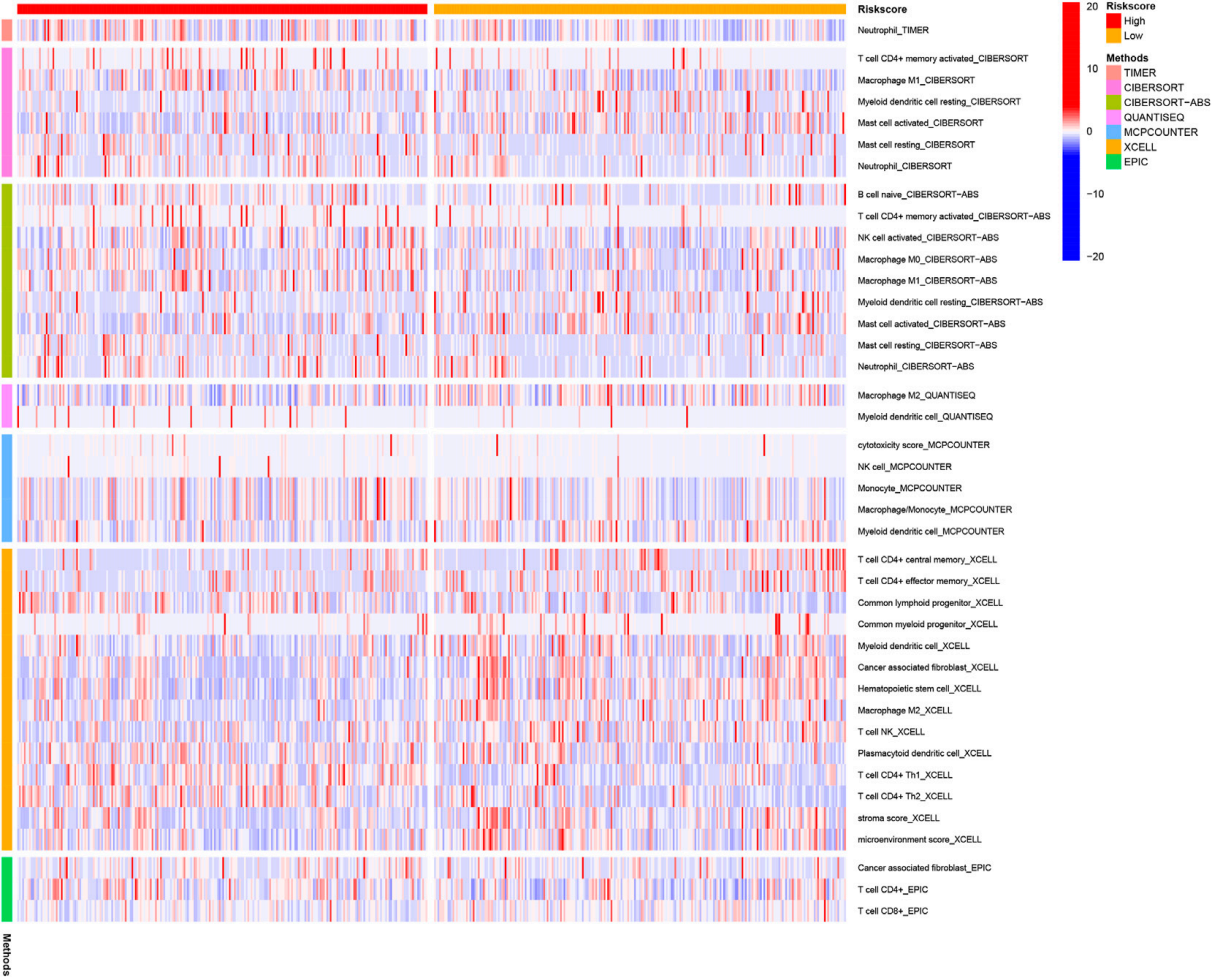
Immune cells' infiltration between high-risk and low-risk groups based on seven kinds of analyses.

DEGs among low- and high-risk groups could be extracted by applying the “limma” R package with standards—FDR < 0.05 and |log2FC | ≥ 1. In total, there were 430 DEGs identified (the data are shown in Supplementary Table S4). The results of the GO analysis indicated that the DEGs were mainly enriched for chromosomal activity and homeostasis (Figure 6A). For exploring the relationships among the risk levels and the immune status in LUAD, the ssGSEA was used to compare the enrichment fractions of 16 kinds of immune cells and the activities of 13 kinds of immune-related pathways in the TCGA database. Interestingly, the low-risk subgroup had higher standards of aDCs, DCs, and iDCs, as did the mast cells. The HLA and type-2 IFN reaction pathway indicated higher activity in the low-risk group, while on the contrary, the MHC class-1 pathway showed higher activity in the high-risk group (Figures 6B,C). Tumor-infiltrating lymphocytes (TILs) and plasmacytoid dendritic cells (pDCs) had the highest correlation in the LUAD immune microenvironment with an R value of 0.86; the mast cells and Tfh were negatively correlated with an R value of −0.04 (Figure 6D). Immune checkpoints and T-cell coinhibition pathways showed the highest positive correlation with an R value of 0.9 (Figure 6E). *WDR4*, *LARP1*, and *NCBP1* were inversely correlated with most immune cells and immune functions (Figure 6F). The relationship between the signature and immune infiltration is displayed in the heatmap according to the analyses of TIMER, CIBERSORT, CIBERSORT-ABS, xCELL, quanTIseq, EPIC, and MCP-counter (Figure 7). The result of CIBERSORT indicated that the proportions of myeloid dendritic cells and active mast cells were higher in the low-risk group, whereas the proportion of neutrophils was higher in the high-risk group.

## Discussion

As the most common histological subtype of lung cancer, LUAD results in a severe incidence rate and death rate (Bray et al., 2018). In this study, we have shown the mRNA standards of 29 m7G methylation–related genes in LUAD tissues and found that 12 of them were differentially expressed when compared with normal tissues. In order to further evaluate the prognostic value of the m7G methylation–related regulators, we used Cox univariate analysis and finally constructed a three-gene risk signature.

m7G is a substantially positively charged modification on the 5′ cap of eukaryotic mRNA and also appears at defined internal locations of tRNA and rRNA (Malbec et al., 2019). In humans, m7G is catalyzed and set up *via* a complex composed of methyltransferase‐like 1/WD repeat domain 4 (METTL1/WDR4) (Boulias and Greer, 2019). METTL1 serves as the m7G catalyzing enzyme but WDR4 acts as a stabilizer (Boulias and Greer, 2019; Zeng et al., 2021). Growing evidence indicates that a variety of disorders and tumors are associated with disturbances in RNA transcriptional modification and translational control (Bray et al., 2018; Obeng et al., 2019). It has been proposed that mutation in *WDR4* causes a unique microcephaly primitive dwarfism by impairing tRNA m7G46 methylation (Shaheen et al., 2015). Recently, increasing evidence have shown that *WDR4* plays a vital role in the occurrence and progression of tumors. An analysis of human pan-cancer based on TCGA, GTEx, and CCLE databases has revealed that except for the TCGA kidney chromophobe cohort, *WDR4* is continually upregulated in most cancer types. Besides, a higher expression level of *WDR4* is an adverse factor in many kinds of cancers, such as LUAD. More crucially, a link between *WDR4* and immune infiltration suggests *WDR4* as a target for immunotherapy in cancer (Zeng et al., 2021).

Accumulating evidence have verified that *WDR4* expression increased the m7G methylation level in tumors, promoting tumor cell proliferation and progression, such as in hepatocellular carcinoma (HCC) (Xia et al., 2021) and neck squamous cell carcinoma (Chen et al., 2022). In addition, *WDR4* can enhance HCC metastasis and chemoresistance by epithelial–mesenchymal transition (EMT) (Xia et al., 2021). Importantly, a study by Ma et al. (2021) observed that in lung cancer samples of humans, *METTL1* and *WDR4* expression levels were significantly raised and negatively related to patient outcomes. Moreover, *METTL1* or *WDR4* depletion inhibited proliferation and invasion, as well as *in vivo* tumor growth of lung cancer cells. Their findings uncovered the oncogenic role of *METTL1/WDR4*-mediated m7G tRNA modification in lung cancer. The conclusions from these prior studies are consistent with the result that high expression levels of *WDR4* are connected with bad clinical outcomes of LUAD, such as in our study.

La-associated protein 1 (LARP1) is an m7G cap-binding protein, belonging to the LARP family and most widely studied RNA-binding protein (RBP) family (Cockman et al., 2020). We found that LARP1 has a highly conserved DM15 region. Crystallographic studies have shown DM15 regionally binds 7-methylguanosine 5ʹ-5ʹ triphosphate (m7Gppp) partial and terminal oligopyrimidine (TOP) mRNA-invariant first cytidine (Lahr et al., 2017). *LARP1* has attracted much attention in recent years due to its effect on the mTOR signaling cascade and TOP mRNA translation. These results suggest opportunities for further pharmacological development. However, the role of LARP1 in this process is controversial. Some scientists believe that LARP1 inhibits TOP translation, while others believe otherwise (Hong et al., 2017; Cassidy et al., 2019). Previous studies have found that LARP1 is overexpressed in most epithelial malignancies, and its expression level is correlated with clinical outcomes. The study by Xie et al. (2013)found that high levels of LARP1 protein in HCC tumor tissues were associated with decreased survival time and an increased risk of 5-year death, tumor size, and Child–Pugh score. These results are consistent with other malignant tumors, such as intrahepatic cholangiocarcinoma (Jiang et al., 2018), cervical cancer (Mura et al., 2015), and ovarian cancer (Hopkins et al., 2016). Importantly, there are some studies on LARP1 and LUAD. A study by Han et al. (2018) found that circ-BANP facilitates lung cancer cell living, proliferation, migration, and invasion by targeting miR-503/*LARP1*. Similarly, Xu et al. (2017) found that in contrast to normal control cells, non–small-cell lung cancer (NSCLC) cells had higher mRNA levels of LARP1. The knockout of LARP1 in NSCLC cells can inhibit cell proliferation, migration, invasion, and tumorigenesis. In further studies, LARP1 was found to be the target of miR-374A. Meanwhile, according to the study (Xu et al., 2017), the XIST/miR-374A/*LARP1* axis is associated with lung cancer. In keeping with previous findings, we found that in LUAD tissues, LARP1 was upregulated, and its high expression means unfavorable living outcomes, showing it has the function of a proto-oncogene. There is a consensus in these studies that LARP1 exerts a key part in LUAD progression and may be a valuable therapeutic strategy. However, whether/how LARP1 participates in LUAD promotion by m7G methylation has not been investigated before and is worthy of further exploration.

Nuclear cap-binding protein subunit 1 (NCBP1) is a kind of heterodimer of nuclear cap-binding proteins, which along with NCBP2 and NCBP3 constitute the nuclear cap‐binding complex (CBC), an important player in the RNA posttranscriptional regulation mechanism (Gebhardt et al., 2015; Dou et al., 2020). It was first discovered that CBC bound to the m7G ′cap structure’ of the new transcription mRNA and controlled downstream RNA biological parent in HeLa cells (Izaurralde et al., 1994; Izaurralde et al., 1995). However, not much is considered as part of *NCBP1* in tumors. *NCBP1* silencing has been reported to suppress the growth of HeLa cells (Gebhardt et al., 2015). In a recent study, it was shown that downregulation of *NCBP1* reduced the proliferation and migration of LUAD cells, while overexpression of *NCBP1* did the opposite (Zhang et al., 2019b). This is consistent with our analysis, the expression of *NCBP1* is positively associated with patient risk, accompanying a poor prognosis. Mechanistically, they found cullin *4B* (*CUL4B*) could be a downstream target gene of *NCBP1* in NSCLC. Unfortunately, this study did not explore whether *NCBP1* affects LUAD cell proliferation through the mechanism of m7G methylation, which needs to be further verified (Zhang et al., 2019b). Our study may provide some insights for further research.

It is becoming increasingly apparent that the degree of malignancy of cancer is jointly determined by its intrinsic characteristics and the TME (Joyce and Fearon, 2015; Jiang et al., 2021; Jiang et al., 2022). TMEs, which include B lymphocytes, T lymphocytes, dendritic cells (DCs), natural killer (NK) cells, and macrophages, influence both the expression of tumor cells and clinical course of cancer (Binnewies et al., 2018; Li et al., 2021b). According to the DEGs among distinct risk groups, we conducted ssGSEA to compare the immunity of the two risk subgroups and unexpectedly discovered that the low-risk group in the TCGA queue had higher fractions of mast cells and DCs. Takahashi et al. (2016) illustrated the beneficial influence of the DC vaccine on the OS in 260 advanced NSCLC patients, suggesting a possible clinical benefit of DCs. However, few research studies have been concerned with the relationship between LUAD and DCs. In this study, we found that high infiltration of DCs was correlated to the low risk of LUAD, which indicates a good prognosis in lung cancer patients, suggesting a therapeutic advantage of activating DCs in the TME. Similarly, high mast cell infiltration has been reported as an indicator of good prognosis in NSCLC, independent of the tumor stage, which can be an explanation for mast cells having had high infiltration in low-risk groups of this study. In our study, we found that a high-risk level was associated with impaired antitumor immunity, containing type II interferon reaction, as well as HLA. In summary, based on this study and our analysis of immunity, we speculate that m7G methylation regulates the immune microenvironment of tumors and poor prognosis in high-risk patients may be related to the weakened antitumor immune ability.

There are few studies on methylation of m7G in tumors, especially on the mechanism of methylation in LUAD. Our research has shown three m7G methylation–related genes in LUAD and preliminarily assessed prognostic value of m7G methylation–related genes. Additionally, a conceptual framework for further investigation is also provided. However, due to the lack of data, we are still unable to clarify the role of these regulatory factors in the m7G methylation pathway in LUAD, and this issue needs further research.

## Conclusion

In summary, this study demonstrates that m7G methylation–related genes are closely connected to LUAD. Moreover, it provides a novel three-gene signature (*WDR4*, *LARP1*, and *NCBP1*) for forecasting LUAD prognosis. M7G modification plays an important role in a variety of tumors, showing great potential in clinical diagnosis and treatment. Its role in chemotherapy resistance and TME remodeling suggests that targeting dysfunctional m7G sites through posttranscriptional editing is combined with chemotherapy or immunotherapy, which makes it possible to obtain better treatment results in the future. m7G modification is a research topic that is still in its preliminary stage, and the important role of m7G in tumor progression and its value in clinical pathology provide potential ideas for the clinical management and treatment of LUAD.

## Data Availability

The original contributions presented in the study are included in the article/Supplementary Material; further inquiries can be directed to the corresponding author.
